# Does respiratory co-infection facilitate dispersal of SARS-CoV-2? investigation of a super-spreading event in an open-space office

**DOI:** 10.1186/s13756-020-00861-z

**Published:** 2020-12-02

**Authors:** Dana Weissberg, Jürg Böni, Silvana K. Rampini, Verena Kufner, Maryam Zaheri, Peter W. Schreiber, Irene A. Abela, Michael Huber, Hugo Sax, Aline Wolfensberger

**Affiliations:** 1 Division of Infectious Diseases and Hospital Epidemiology, University Hospital Zurich, University of Zurich, Zurich, Switzerland; 2 Institute of Medical Virology, University of Zurich, Zurich, Switzerland; 3 Department of Internal Medicine, University Hospital Zurich, University of Zurich, Raemistrasse 100, 8091 Zurich, Switzerland

**Keywords:** COVID-19, SARS-CoV-2, Super-spreading, Co-infection, Adenovirus, Transmission, Secondary attack rate, Outbreak investigation

## Abstract

**Background:**

Super-spreaders are individuals infecting disproportionately large numbers of contacts. They probably play a crucial role in the transmission of severe acute respiratory syndrome coronavirus 2 (SARS-CoV-2). We describe a super-spreading event within a team working in an open-space office and investigate factors potentially having facilitated SARS-CoV-2 transmission.

**Methods:**

In this retrospective cohort study, semi-structured telephone interviews with all team members were carried out to identify symptoms, contacts, and adherence to basic hygiene measures. During site visits, we gathered information about workplace and seating arrangements. The secondary attack rate in office and households was calculated. Potential respiratory viral co-infections were assessed by multiplex PCR. SARS-CoV-2 whole-genome sequencing was performed using a tiled-amplicon sequencing approach.

**Results:**

Of 13 team members, 11 fell ill with Coronavirus disease 2019 (COVID-19). Due to the sequence of events and full genome sequence data, one person was considered the index case for this outbreak, directly infecting 67 to 83% of the teammates. All team members reported repetitive close contacts among themselves during joint computer work, team meetings and a “Happy Birthday” serenade. Two individuals shared nuts and dates. The arrangement of the office and meeting rooms precluded sufficient adherence to physical distancing. The index case and a further individual were diagnosed with an adenovirus serotype 4 co-infection.

**Conclusion:**

We identified several environmental and behavioral factors that probably have facilitated the transmission of SARS-CoV-2. The relevance of the adenovirus co-infection remains unclear and merits further investigation.

## Background

The Coronavirus disease 2019 (COVID-19) emerged in late December 2019 in Wuhan, China. The causative agent is the severe acute respiratory syndrome coronavirus 2 (SARS-CoV-2), a betacoronavirus, related to SARS-CoV and Middle Eastern respiratory syndrome coronavirus (MERS-CoV). COVID-19 was declared a pandemic in March 2020 [1]. As of November 13, more than 53,000,000 of COVID-19 cases have been reported worldwide [2]. The primary mode of transmission is assumed to happen by droplet or contact [3, 4]. It has not yet been resolved whether SARS-CoV-2 might also spread through aerosols [5]. Since highest viral loads in throat swabs are present at the time of symptom onset, peak infectiousness was inferred to be on or before symptom onset [6]. An infectiousness model presented by Ferretti et al. suggests that pre-symptomatic and symptomatic individuals cause between a third and half of all transmissions, respectively, whereas continuously asymptomatic infected individuals and environmental transmission play a minor role [7].

At the beginning of the pandemic, the basic reproductive number R_0_ for COVID-19 in Wuhan was reported to be around 2.0 by several authors [8–10]. However, the distribution of individual R values was described to be highly over-dispersed, with 80% of infections being caused by less than 10% of cases [11, 12]. This finding is in line with the concept of the empirical 20/80 rule – suggesting that 20% of individuals contribute to at least 80% of transmission potential. This rule was based on observational and modeling studies more than 20 years ago [13], and many host-pathogen interactions were later found to follow this rule [14].

Individuals disproportionately infecting a large number of others with a pathogen are called “super-spreaders”. Super-spreading events were described in transmission events during the SARS-CoV epidemic in 2003 and also for MERS-CoV [15–18]. In COVID-19, such events were reported in hospitals, in choir rehearsals, holiday visits, and restaurant meals [19–23]. Here, we report a cluster of eleven COVID-19 cases in a thirteen-person team working in an open-space office, taking place in the canton of Zurich, Switzerland, with onset on March 10, 2020. By this day, the total number of confirmed COVID-19 cases in the canton of Zurich was 88, corresponding to overall 0.06 cases per 1000 inhabitants [24]. Prevailing recommendations of the Swiss Federal Office of Public Health for employees included social distancing, office-splitting, home office and hand hygiene, however, wearing of face masks was not advised. We conducted a retrospective cohort study and outbreak investigation to assess secondary attack rates and investigate factors that might have facilitated SARS-CoV-2 transmission.

## Methods

### Epidemiological investigation

We conducted a retrospective outbreak investigation and cohort study of a cluster of COVID-19 cases in a team of 13 members performing desk work in an open-space office. Semi-structured telephone interviews with all team members were carried out to obtain data about demographics, work attendance, activity patterns, contact with team and household members, adherence to hygiene measures, and clinical symptoms. Interviews were audiotaped after informed consent was obtained. During site visits in the open-space office information about workplace and seating arrangements and the ventilation system was obtained.

### Definitions

A confirmed case of COVID-19 was defined as any individual with a respiratory sample positive for SARS-CoV-2 using a laboratory-based PCR test, or any individual with symptoms suggestive for COVID-19 with an epidemiological link to a PCR-positive individual and an individual in whom SARS-CoV-2-specific antibodies were detected. A probable case of COVID-19 was defined as any individual with symptoms suggestive for COVID-19 having an epidemiological link to a confirmed case. The day of symptom onset was defined as the day when any first symptom occurred, including non-specific symptoms such as fatigue.

The potential infective period was defined as 48 h before to 10 days after symptom onset or 48 h after symptom cessation, whatever occurred later. The incubation period was calculated as the mean time from first to last exposure until development of symptoms. A high-risk contact was defined as a face-to-face contact with a COVID-19 case within 2 m and longer than 15 min, or direct contact with respiratory secretions of a COVID-19 case. Secondary attack rates were calculated as number of cases divided by number of contacts. Fisher’s exact test was used to calculate 95% confidence intervals. For the “office attack rate” exclusively confirmed cases were considered, for the “household attack rate” probable cases were also included.

### SARS-CoV-2 PCR and serology

Diagnostic testing for SARS-CoV-2 was performed through RT-PCR of a nasopharyngeal swab as described by Corman et al. [25]. Cycle threshold (Ct) values of the E gene target were obtained from the first diagnostic PCR. Nasopharyngeal swabs were tested for respiratory viruses other than SARS-CoV-2 by the CE-IVD marked FTD Respiratory Pathogens 21 Multiplex-PCR (Fast-track Diagnostics, Siemens Healthineers, Erlangen, Germany). This panel simultaneously detects influenza A/B, respiratory syncytial virus A/B, non-SARS Coronaviridae (NL63, 229E, OC43, HKU1), parainfluenza virus 1–4, metapneumovirus A/B, bocavirus, adenovirus, rhinovirus/enterovirus, parechovirus and *Mycoplasma pneumoniae*. This test was performed in 9 team members with sufficient sample material available (E1-E5 and E8-E11). SARS-CoV-2 serologic testing was performed using an in-house developed bead-based antibody assay using Luminex technology that detects IgG, IgA and IgM antibodies against subunit 1 (S1), subunit 2 (S2) of the spike protein and nucleoprotein (NP) of SARS-CoV-2 in individuals after a minimum of 14 days after symptom onset or a minimum of 60 days after exposure in non-symptomatic individuals.

### SARS-CoV-2 genome sequencing and analysis

SARS-CoV-2 whole-genome sequencing was performed using a previously described tiled-amplicon sequencing approach [26, 27]. Total nucleic acids were extracted followed by reverse transcription with random hexamers and oligo-dT priming (ratio 3:1) using SuperScript IV Reverse Transcriptase (Thermo Fisher Scientific) [28]. The generated cDNA was used as input for 14 overlapping PCR reactions (ca. 2.5 kb each) spanning the viral genome using Platinum SuperFi DNA Polymerase (Thermo Fisher Scientific). Amplicons were pooled per patient before NexteraXT library preparation and sequencing on an Illumina MiSeq for 1 × 151 cycles. To generate SARS-CoV-2 consensus sequences, all reads were iteratively aligned using SmaltAlign (github.com/medvir/SmaltAlign). Sequences were uploaded to GISAID (accession numbers EPI_ISL_508,864 to 508,864; 509,222). Multiple sequence alignment was done with MAFFT v7.271 [29], followed by phylogenetic analysis using RAxML [30].

### Ethical approval

The Zurich Cantonal Ethics Commission waived the necessity for a formal ethical evaluation based on the Swiss law on research on humans (Req-2020-00324).

## Results

### Team and workspace

**Fig. 1:**
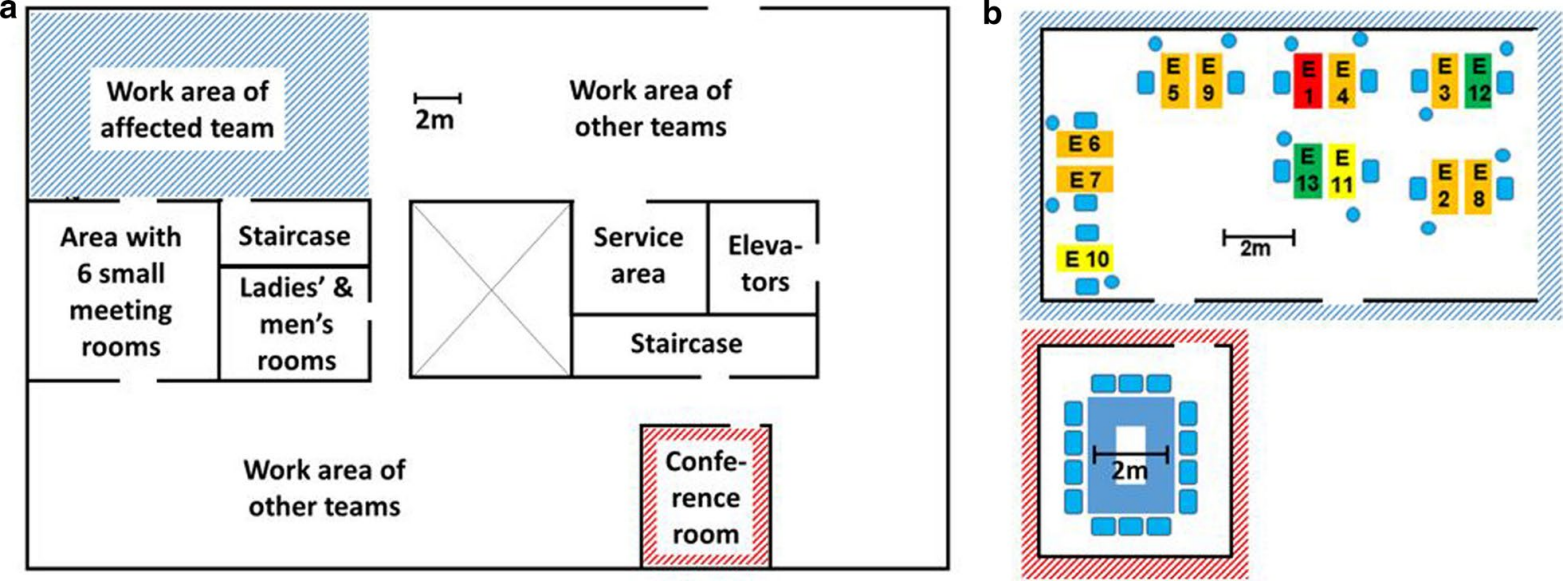
Floor plan and seating arrangement. **a**: Floor plan; **b**: Seating arrangement of affected team in the work area and in the conference room. Large coloured rectangles = desks (red: desk of the index case; orange: desks of team members with COVID-19 who fell ill until March 14; yellow: desks of team members with COVID-19 who fell ill after March 14; green: desks of team members who did not fall ill; blue: desks in conference room); Small blue rectangles = seats; Small blue circles = side seats

The team consisted of 13 individuals with a median age of 49 years (IQR, 44–55). The workplace is located in a 700m^2^ sized open-plan office, approximately one quarter of the office is occupied with the concerned team (Fig. 1). A service area is located in the middle of the office for individual consumption. Restrooms are shared with other teams on the same floor. Each team member owns a personal workstation with desk and computer, and workstations are a minimum of 0.8 m apart. A 30m^2^ conference room and several smaller meeting rooms of 7m^2^ or 9m^2^ are available. The ventilation system provides air renewal within 1 hour in the open office space, and within 15 min in the conference room, respectively. There is no recirculation of air or strong directed airflow. Windows can be tilted, but not completely opened.

### Description of COVID-19 outbreak

During 13 interviews with a median duration of 37 min we obtained the following information: On March 10, employee E1 experienced symptoms of fatigue and minimal cough at noon and left the office early in the afternoon. The following day, he informed the company’s medical officer about his condition and was isolated at home. He was not tested as he did not fulfill the testing criteria for SARS-CoV-2 at that time. On March 13 – by then his respiratory symptoms had worsened and he reported rhinorrhea, sore throat, and fever – he was informed that his friend, with whom he had close contact on a party on March 7, had tested positive for SARS-CoV-2.

Two days after E1’s last presence in the office, in the late evening of March 12, his teammate E2 developed cough and did not attend work the next day. On March 13, six other team members (E3, E4, E5, E6, E7, and E8) started feeling unwell; E3, E5 and E8 stayed home, E6 developed prodromal symptoms in the afternoon while at work, and E4 and E7 developed first symptoms after the end of their workday. An eighth team member, E9, fell ill on March 14. RT-PCRs for SARS-CoV-2, performed between March 14 and March 16, resulted positive for all employees E1-E9. Due to the sequence of events, E1 was considered the index patient for this outbreak. Home isolation and quarantine measures were imposed on March 15 for all sick and yet healthy team members, respectively.

On March 18, 8 days after onset of symptoms of E1, team member E10 fell ill, and was tested positive for SARS-CoV-2 the next day. On March 19, team member E11 came down with fever and cough. E11 had already been tested negative on March 15 while being asymptomatic, and no SARS-CoV-2 PCR was performed after symptom onset. However, a serology taken from E11 23 days after symptom onset resulted positive for SARS-CoV-2-specific IgA, IgG and IgM. Therefore, 11 of 13 team-members were considered confirmed COVID-19 cases. Two team members (E12 and E13) remained asymptomatic throughout the 2 weeks after last exposure and were tested seronegative 9 weeks after exposure. Symptoms of the team members are shown in Table 1.

**Table 1:** Clinical presentation of COVID-19 in affected team members

Symptoms	n (%)
Fever	11 (100%)
Cough	10 (91%)
Fatigue	8 (73%)
Myalgia	6 (55%)
Dysgeusia	6 (55%)
Headache	5 (45%)
Sore throat	2 (18%)
Chest pain	2 (18%)
Back pain	2 (18%)
Diarrhea	1 (9%)

### Team interactions and adherence to hygiene measure

**Fig. 2:**
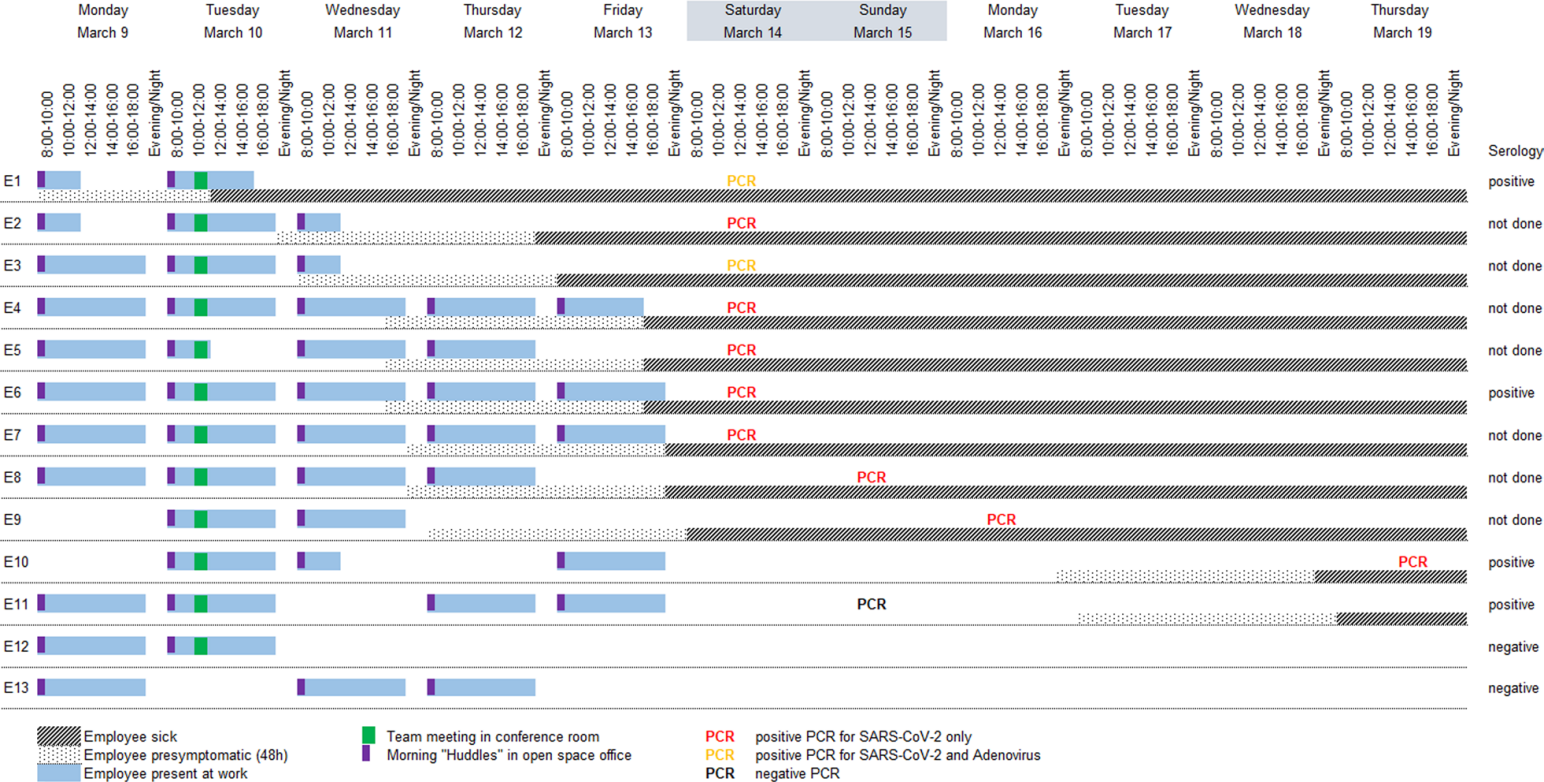
Transmission chain of COVID-19. Abbreviations: h, hours; PCR, polymerase chain reaction

Physical presence of the individual team members in the office is depicted in Fig. 2. Every morning, the team performs a five-minute ‘huddle’ team meeting, standing close to each other between their workstations. Working together at the same workstation, including sharing of mouse and keyboard, is frequent in this team. For this purpose, an extra chair is available at every desk. All team members reported to have had high-risk contacts with several other team-members in front of the computer throughout the week, but contacts could only rarely be reconstructed in detail, except that E1 specifically reported to have had frequent and close contact with E8 and E9 on March 10, while being introduced into new processes. Lunch breaks are usually spent among team members in the canteen, however, E1 did not join his teammates on March 9 and 10. Shared coffee breaks are not common, but E5 reported to have eaten nuts and dates from the same bowl as E1 on March 9 and 10.

On March 10, a one-hour team meeting took place in the conference room (Fig. 1) shortly before first symptoms developed in E1. During the meeting, E2 remembered sitting next to the index case. Noteworthy, the participants sang “Happy Birthday” to celebrate a team members birthday (not E1’s birthday), but no hugs or kisses were exchanged. E5 joined the meeting later and was not present during singing. In summary, E1 spent approximately 3 h at work while having first prodromal symptoms.

On Friday 13, E4 and E10 had a meeting in the late afternoon, and E11 reported high-risk contact to almost all present team members. At this day, E6 had first prodromal symptoms for approximately 2 h before leaving work. From March 16, all members of the team stayed home for isolation or quarantine.

During working hours, none of the team members was wearing a face mask or consistently maintained a physical distance (> 2 m) during personal interactions and team meetings. Six (46%) employees disinfected their hands on occasional or frequent basis, and adherence to respiratory etiquette was self-reported by 62% of all team-members, including the index case.

Except E1, no other sick employee reported private contact with a person having symptoms compatible with COVID-19 within the 2 weeks before symptom onset or before quarantine.

### Viral co-infections and estimation of SARS-CoV-2 viral loads

The Ct value of E1’s SARS-CoV-2 PCR in a swab 4 days after symptom onset was 20.7, corresponding to approximately 10E8 virions/ml. Median Ct values of the other team members was 21.5 (IQR: 19.9–24), collected a mean of 1.3 days after symptom onset. To assess the presence of viral co-infections, the available nasopharyngeal swabs of nine team members were re-analyzed by multiplex PCR. Co-infection with adenovirus was found in the index patient E1 and in E3, genotyping of E1’s isolate revealed adenovirus E Serotype 4 (AdV-4). Sequencing of E3’s isolate was not performed.

### Phylogenetic analysis

For eight of the eleven individuals, the full-length genome could be sequenced (for E6, E7 and E11 no material was available). Phylogenetic analysis showed that all sequences form a cluster within lineage 20A of SARS-CoV-2 (nextstrain nomenclature) [31]. Six of the sequences are identical, while E10 and E2 are each one nucleotide different from the others (Additional file 1). Sequences of several other individuals from Switzerland not related to this outbreak, sampled before March 9, 2020, have identical sequences.

### Incubation period and secondary attack rate

Assuming that the index patient E1 passed the SARS-CoV-2 to all ten team members (scenario 1), the secondary office attack rate caused by E1 was 83% (95%CI: 52–98). Assuming that E10 and E11 were infected by other team members (scenario 2), the secondary office attack rate caused by E1 was 67% (95%CI: 35–90). The mean incubation period was 4.7 (95%CI: 3.2–6.2) and 4.1 (95%CI: 3.5–4.7) days for scenario 1 and 2, respectively. The eleven employees with COVID-19 had 18 household contacts, of whom 11 were adults (Table 1). Secondary household attack rate of all eleven team members was 39% (95%CI: 17–64) overall, or 55% (95%CI: 23–83) in adult household members. In the two employees co-infected with adenovirus household attack rate was 1/4 (25%), while it was 6/14 (43%) in households of non-co-infected employees.

## Discussion

We report an outbreak of COVID-19 in a 13-person team doing desk work in an open-space office. The temporal occurrence of COVID-19 cases and full genome sequence data suggest that the index case E1 acted as a super-spreader infecting eight or potentially ten of twelve teammates. The index case spent approximately 8 h in the pre-symptomatic and 3 h in an oligosymptomatic state with the team. Behavioral factors such as close team interactions with joint computer work and a one-hour team meeting including singing of a ‘Happy Birthday’ serenade, and environmental factors such as a congregation in a 30m^2^ conference room might have propelled virus transmission. The co-infection of the index person with AdV-4, which was transmitted to one other team member, is noteworthy. Our report demonstrates high transmissibility of SARS-CoV-2 and the possibility of super-spreading events in office co-workers.

We found a secondary office attack rate caused by the index case of 67% when including employees with an incubation period of up to 5 days as secondary cases, or 83% when also including the two workmates with an incubation period of 8 and 9 days. We consider both scenarios possible, as the median incubation period has been reported around 4 to 6 days [8, 11, 32], with a 97.5%CI of 11 to 12 days [32, 33]. As the mutation rate of SARS-CoV-2 is known to be very low and most of the sequences of the reported cluster were identical, conclusive identification of a transmission chain was not possible by phylogenetic analysis. The secondary attack rate we discovered in this place of work is high even in comparison to household transmissions - a setting with hypothetically closer person to person contact compared to work settings - where secondary attack rates of 5 to 20% have been described [8, 11, 34–37]. Literature describing secondary attack rates related to one index-case in other than household settings is scarce. Some rare publications describe secondary attack rates of similar dimensions, i.e. in tourists sharing a holiday chalet (secondary attack rate, 75%) and in people attending a choir rehearsal (secondary attack rate, 87%) [19, 23]. Reasons for super-spreading events and the heterogeneity within the ability of infecting others remain unclear. In theory, environmental factors such as crowding or building ventilation, as well as host- or pathogen-related contributions have been discussed – and often more than one factor plays a role [14, 38].

From the information gathered in the interviews with team members, we believe that host factors, specifically behavioral factors of all team members contributed to the outbreak within this team. Interactions during joint computer work lead to proximity, a prerequisite for droplet transmission, and eating nuts from the same bowl as the index case facilitates indirect contact transmission. The one-hour team meeting including singing a “Happy Birthday” serenade probably has also fueled transmission of SARS-CoV-2 as several super-spreading events have been reported from choir rehearsals [23]. Correspondingly, one of the two unaffected team members did not join the specific meeting. We were not able to identify other non-behavioral host-specific risk factors. It would have been interesting, though, to further assess physiological factors of the index case, such as particle emission during speech, as the concept of speech super-emitters was postulated by Asadi et al. [39].

We also consider environmental factors relevant for this open space office outbreak. Employees’ desks are arranged close to each other and the team meeting took place in a confined conference room with eleven people enclosed in 30m^2^, precluding the suggested minimum distance between people of > 2 m. Prolonged, unprotected exposure in closed congregations were already described to be a risk factor for SARS-CoV-2 transmission [40, 41]. On the other hand, we do not have evidence that the ventilation system played an active role in this outbreak. In comparison to other settings where air condition was suspected to facilitate transmission [22], there is no strong directed airflow in the rooms of this open space office and also no air recirculation. Since it is not yet clear if aerosols might be involved in the transmission of SARS-CoV-2, we cannot formally rule out, though, that a higher air change rate might have been beneficial to lower transmission risk, especially after droplet- and aerosol-generating procedures such as singing.

Pathogen-related factors might have also played a role in this cluster. The index case E1 had a relatively high viral load of 10E8 4 days after symptom onset. Viral loads of SARS-CoV-2 were observed to be highest at the time of symptom onset, and infectiousness was inferred to peak on or before symptom onset [6]. Therefore, we hypothesize an even higher viral load in the hours before or at the beginning of symptom onset – a time E1 spent with office colleagues. Additionally, the index case was co-infected with an adenovirus. AdV-4 has caused several outbreaks of febrile respiratory infections in civilian and military populations [42]. Viral co-infections were described to facilitate dispersal of different bacterial pathogens and were hypothesized to have facilitated a super-spreading event of SARS-CoV transmissions in 2003 [43]. Sherertz et al. found that rhinovirus infection leads to high dispersal of *Staphylococcus aureus* in the air even in the absence of coughing or sneezing [44]. The mechanism for this effect still is unclear, but may be caused by swelling of the nasal turbinates and narrowing air passages, in turn leading to a higher speed of the turbulent air flow, which could create air-dispersed infectious particles [44]. The adenovirus of the index case was transmitted to only one other team member. To systematically evaluate the relevance of respiratory co-infections in occurrence of SARS-CoV-2 transmission higher sample sizes would be needed.

Our study has limitations. First, an imperfect or biased recall could have affected the accuracy of the data such as information about contacts and the exact time of symptom-onset. For example, the seating arrangements during the team meeting and information about occurrence and duration of person-to-person interactions could not be recalled in detail. This, in turn, prevented the identification of perspicuous reasons for not falling sick of the two team members E12 and E13. Second, exposure of teammates to one or several other index persons other than E1 cannot be excluded, as there are other identical sequences from Switzerland sampled before this super-spreading event. However, in view of the very low total number of confirmed COVID-19 cases in the canton of Zurich, Switzerland, at the time of the described outbreak and the close temporal occurrence of cases, we consider transmission within this team highly likely [24]. Third, most of the team member’s household contacts did not seek testing to confirm COVID-19 as the cause of their illness. It is therefore possible that they had another respiratory disease and were falsely classified as probable COVID-19 cases.

In summary, we describe a COVID-19 cluster caused by a super-spreading event in an open-space office and were able to identify several behavioral and environmental factors propelling transmission. High-density work environments with close person-to-person contact are common worldwide. As reports about super-spreading events in open space offices are scarce, we assume them to be the exception rather than the rule and that either the combination of several factors was relevant for this event or that a specific, still not clearly recognizable factor played a crucial role. Singing might have been relevant, as this is an extraordinary activity for workspaces and was reported to have facilitated transmission in other settings. Host specific physiological factors could have also played a role, but they are inherently difficult to evaluate, especially retrospectively. And the adenovirus co-infection might have acted synergistically. Further research is needed to investigate a potential causal relationship of co-infections and super-spreading events in COVID-19. Early identification of potential super-spreaders or factors facilitating super-spreading events are of great importance for controlling the dissemination of COVID-19. Until more is known about risk factors for SARS-CoV-2 super-spreading, we suggest to strictly follow the rules on hygiene, social distancing, and wearing a mask if distancing is not feasible at all workspaces, especially in open-space offices.

## Supplementary information

**Additional file 1**. Phylogenetic analysis. Phylogenetic tree of the super-spreading event and all high-quality sequences from Switzerland with collection date on or before March 9, 2020 available on GISAID by August 5, 2020. Sequences from the super-spreading event described here are shown in bold.

## Data Availability

The datasets used and analysed during the current study and the raw sequencing reads are available from the corresponding author on reasonable request. The SARS-CoV-2 sequences are available on GISAID (accession numbers EPI_ISL_508864 to 508864; 509222).

## Abbreviations

AdV-4: Adenovirus E Serotype 4
Ct: Cycle threshold
COVID-19: Coronavirus disease 2019
MERS-CoV: Middle Eastern respiratory syndrome coronavirus
SARS-CoV-2: Severe acute respiratory syndrome coronavirus 2
