# Supplementary figures and images for: Does respiratory co-infection facilitate dispersal of SARS-CoV-2? investigation of a super-spreading event in an open-space office
Source: Antimicrob Resist Infect Control. 2020 Dec 2;9:191. doi: 10.1186/s13756-020-00861-z (PMC7708893; doi:10.1186/s13756-020-00861-z)

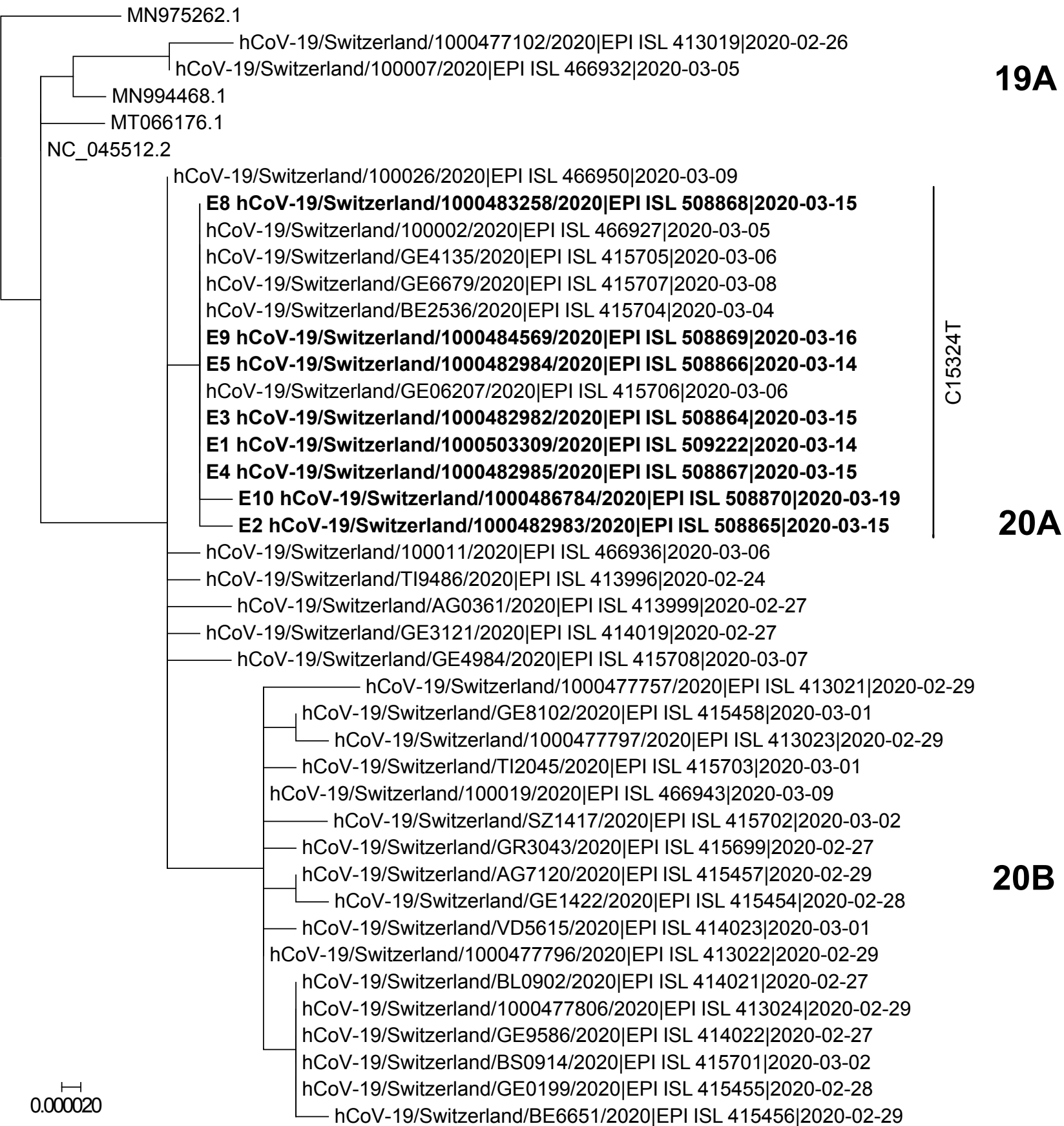

Supplement: Supplementary file 1 — Additional file 1. Phylogenetic analysis. Phylogenetic tree of the super-spreading event and all high-quality sequences from Switzerland with collection date on or before March 9, 2020 available on GISAID by August 5, 2020. Sequences from the super-spreading event described here are shown in bold. [file 13756_2020_861_MOESM1_ESM.pdf]
